# Supplementary material for: Multiplexed lipid nanoparticle barcoding reveals tissue-dynamic kinetic insights and enriched cellular tropism in hepatic zones
Source: Nat Commun. 2026 Jan 7;17:1345. doi: 10.1038/s41467-025-68103-7 (PMC12873177; doi:10.1038/s41467-025-68103-7)
Supplement: Supplementary file 1 — Supplementary Information [file 41467_2025_68103_MOESM1_ESM.pdf]

Supplementary Information

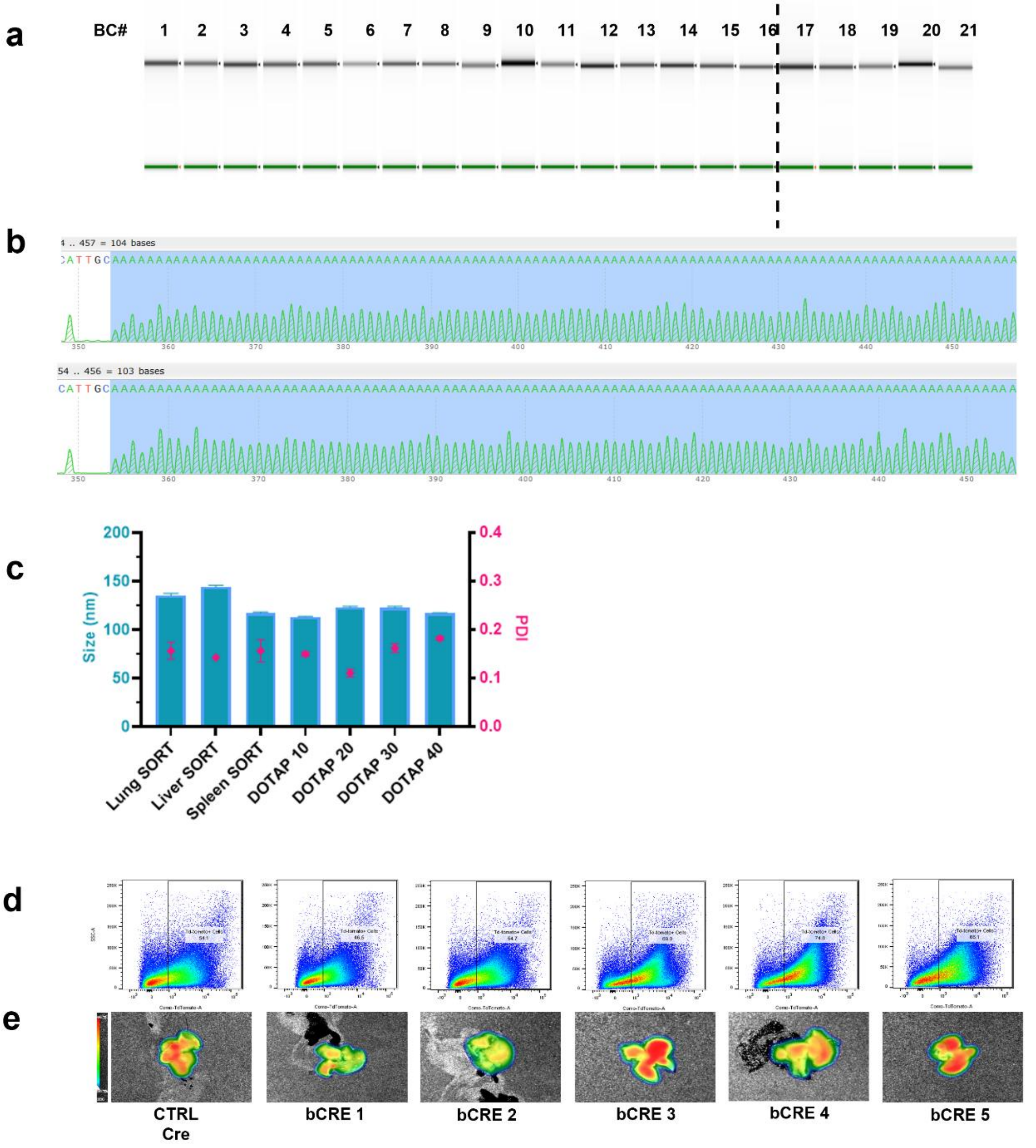

Supplementary Figure 1 │ Barcoded Cre mRNA quality control (a) Tapestation simulated gel image of all 21 barcoded Cre mRNAs used in this study **(b)** Sanger sequencing of selected IVT template to confirm the presence and approximate length of the polyA tail. **(c)** Dynamic light scattering analysis of LNPs used throughout the study reveals consistent size and low dispersity **(d)** Flow cytometry demonstrates that reporter expression from barcoded mRNAs is comparable to non-barcoded counterparts. **(e)** Ex vivo liver images show comparable reporter signal following mRNA delivery between barcoded and non-barcoded Cre mRNAs. .

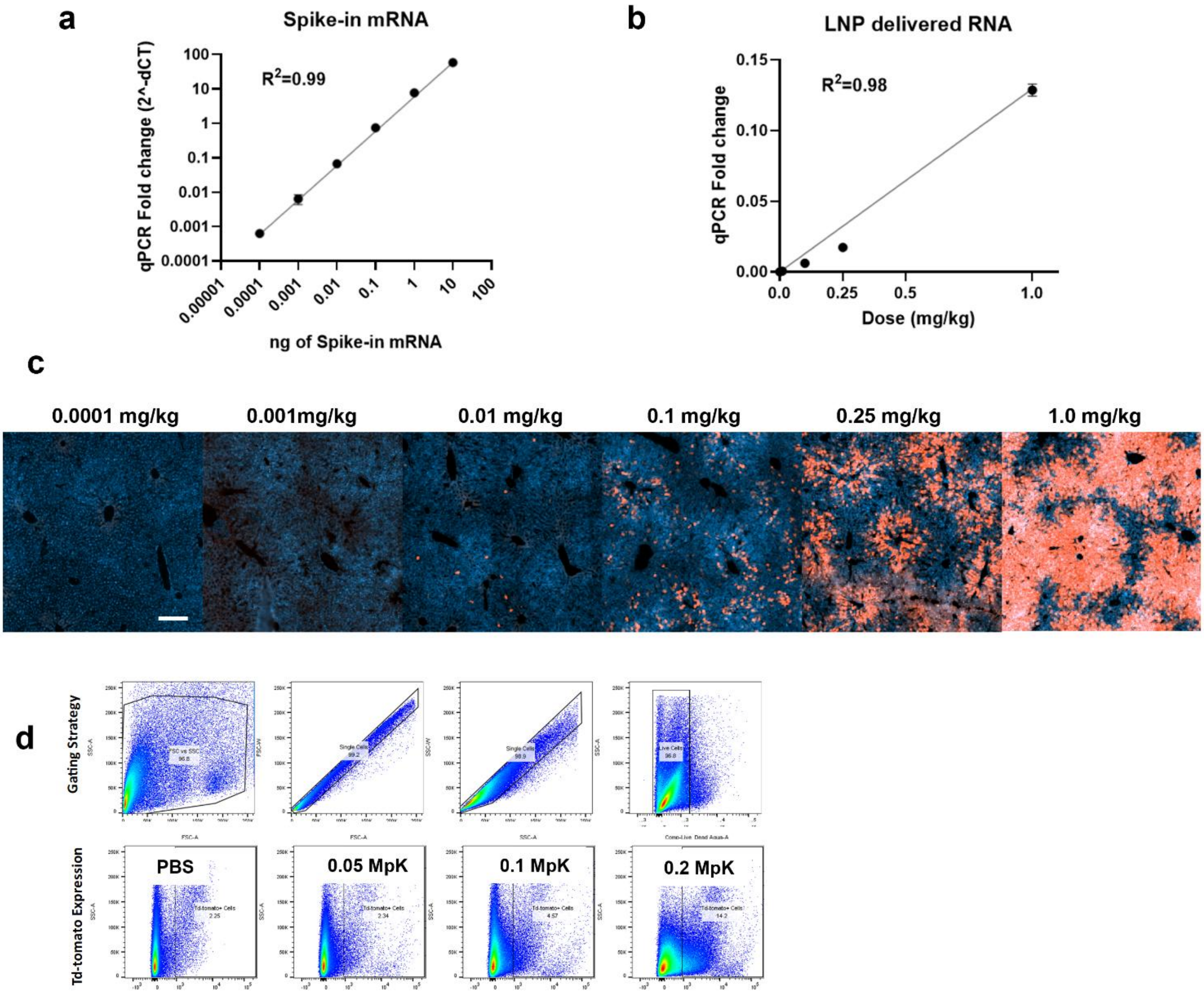

Supplementary Figure 2 │ Standardization and dose-response analysis of liver-targeting LNP formulations. (a) A standard curve generated by spiking known masses of barcoded mRNA into total RNA, followed by reverse transcription and qPCR, reveals a linear relationship between spike-in RNA mass and qPCR fold change. **(b)** A dose-response experiment using 0.0001–1.0 mg/kg of barcoded mRNA-loaded LNPs shows a strong linear correlation between administered dose and liver qPCR signal at 24 hours post-injection. **(c)** Reporter fluorescence images from livers collected in the dose-response study. Scale bar: 200 uM **(d)** Independent dose-response assessment using flow cytometry to quantify reporter expression

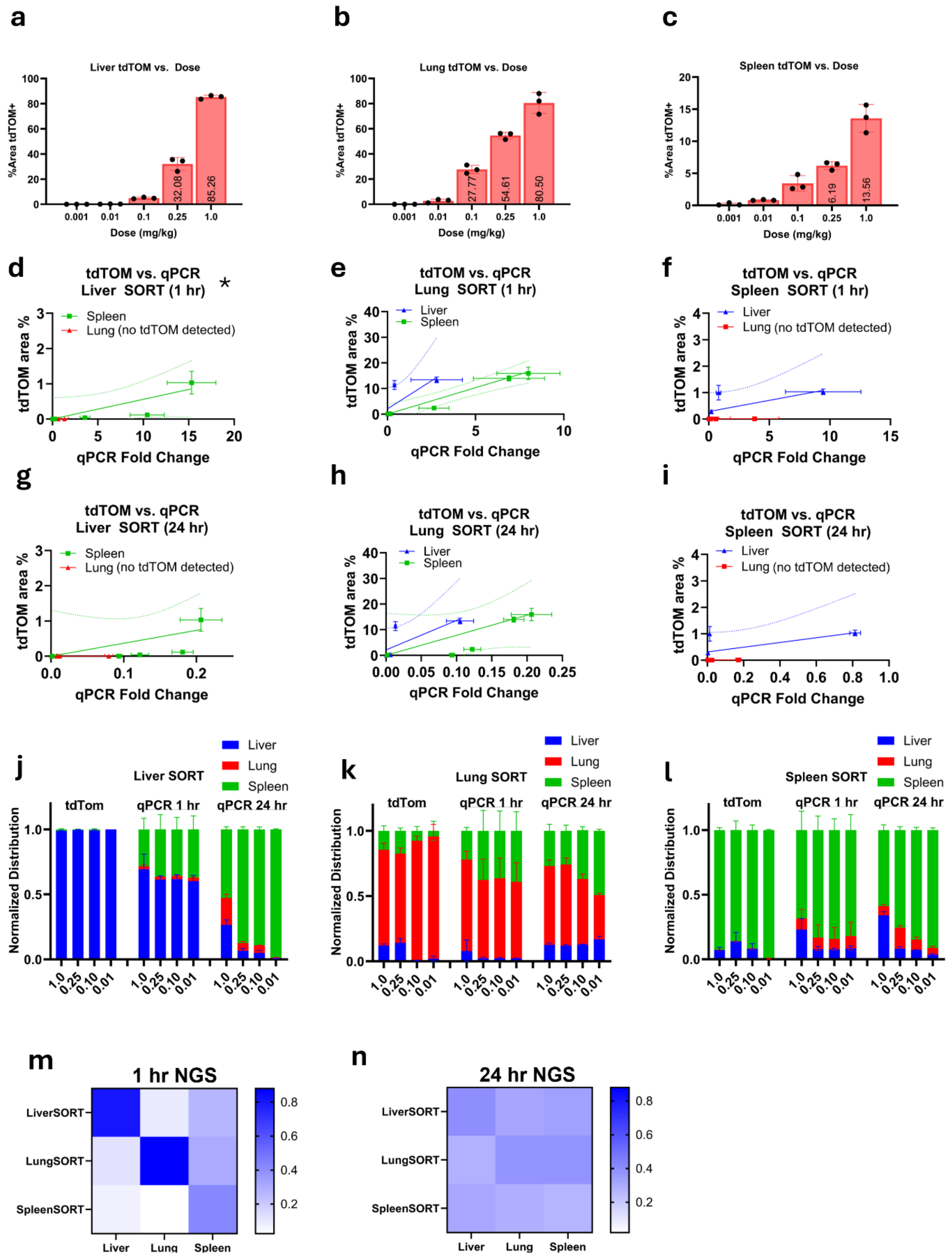

Supplementary Figure 3 │ Comparative biodistribution and reporter correlation of tissue-targeting SORT LNPs. (a-c) tdTom reporter quantification from mice that received various doses of Cre mRNA packaged in Liver SORT **(a)**, Lung SORT **(b)**, or Spleen SORT **(c)** LNPs. Mean +/- SEM **(d-i)** tdTom reporter signal plotted against qPCR fold changes in off-target organs at 1 h or 24 h following administration of Liver SORT **(d, g)** Lung SORT **(e, h)**, and Spleen SORT **(i, l). ***Did not pass homoscedasticity test. **(j-l)** Biodistribution profiles at multiple doses for Liver SORT **(j)**, Lung SORT **(k)**, and Spleen SORT **(l)** LNPs; early timepoints align more closely with observed reporter expression. Mean +/- SEM **(m-n)** Heatmap summarizing a pooled barcoding experiment in which

barcoded Liver, Lung, and Spleen SORT LNPs were formulated with unique barcodes, administered as a pool at 0.1 mg/kg, and harvested at 1-hour **(m)** or 24-hours **(n)**. Target organ enrichment was markedly higher at 1 h for each formulation, while enrichment declined substantially by 24 h.

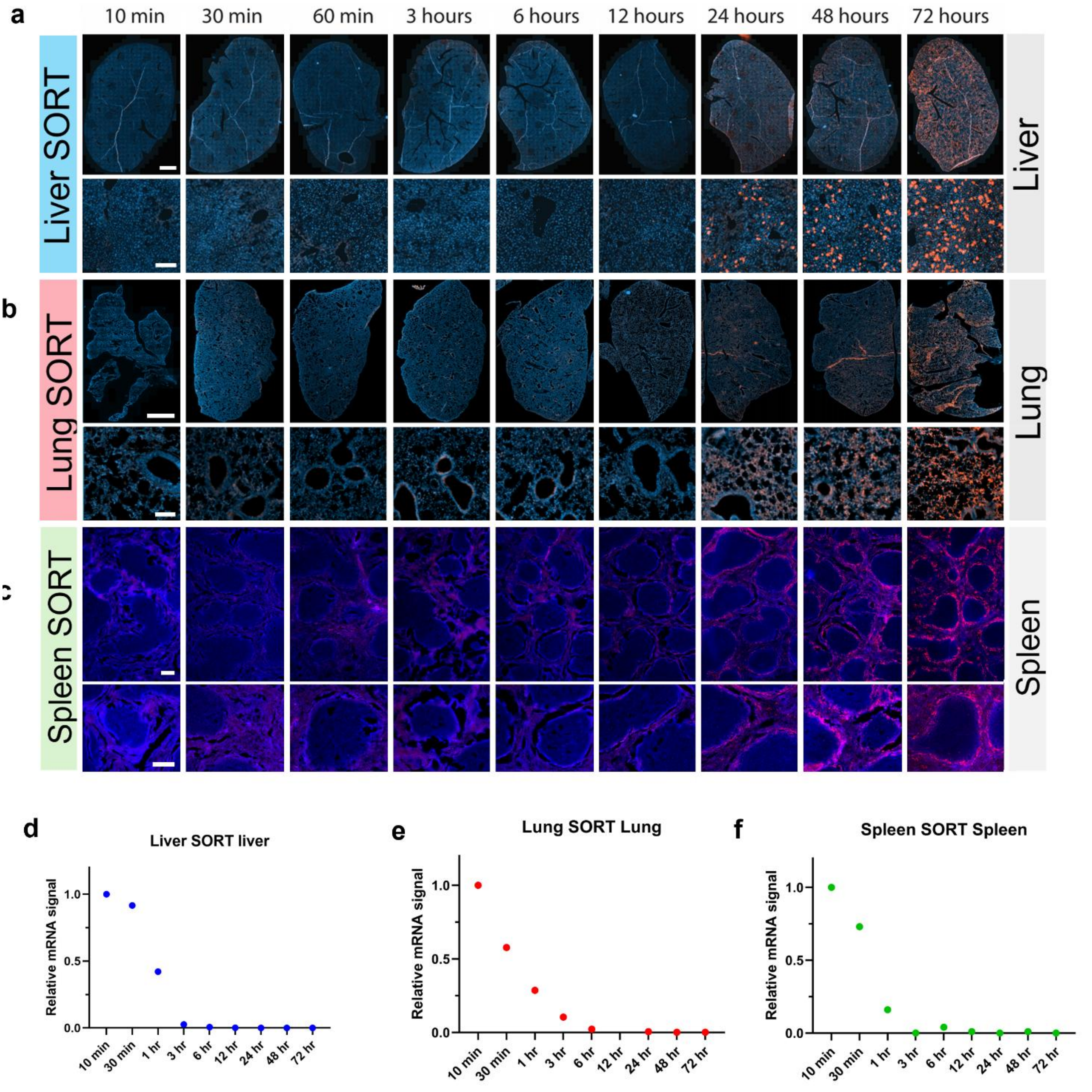

Supplementary Figure 4 │ Reporter fluorescence timeline and mRNA kinetics (a-c) Endogenous fluorescence micrographs following administration of the noted formulations at 0.1 mg/kg of mRNA and collected at the noted timepoints in the (**a**) liver, scale bars: 2mm (top) 200 uM (bottom) (**b**) lung, scale bars: 2mm (top) 200 uM (bottom), and (**c**) spleen, scale bars: 200 uM (both) **(d-f)** qPCR performed on cDNA reverse transcribed from total RNA extracts of liver, lung, and spleen following administration of the noted formulations at 0.1 mg/kg of mRNA.

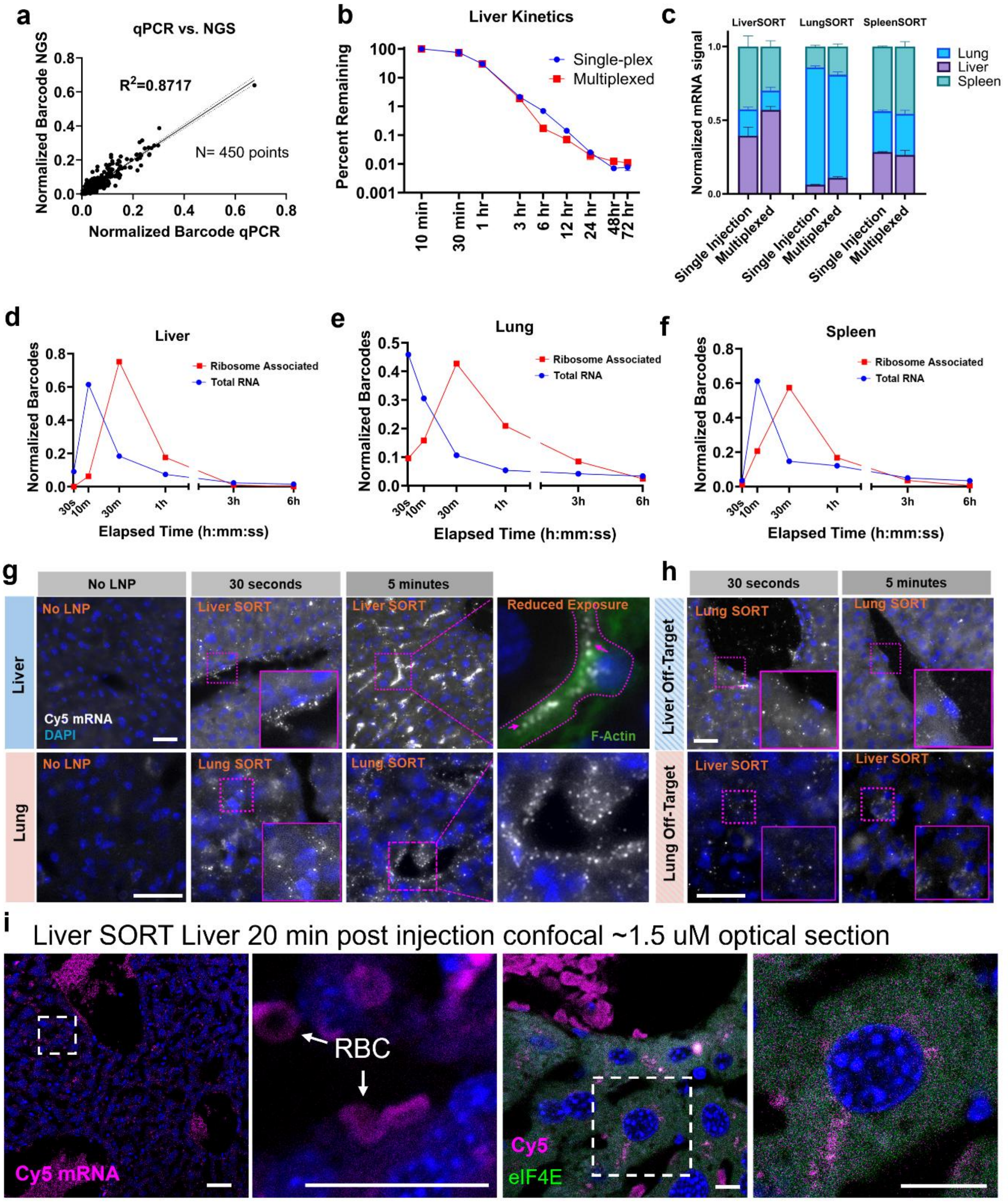

Supplementary Figure 5 │ Validation and imaging of barcode readouts and early LNP biodistribution. (a) Barcode abundance measured by qPCR strongly correlates with values obtained by next-generation sequencing (NGS). **(b–c)** Multiplexed and single-injection kinetic studies at matched timepoints yield comparable barcode profiles and similar biodistribution patterns for liver-, lung-, and spleen-targeting LNPs. **(d–f)** Mulitplexed barcode kinetics experiment showing time coded barcodes associated with total RNA or ribosome profiled RNA. Early biodistribution and enrichment can result in functional delivery **(g-h)** Fluorescent micrographs showing the distribution of Cy5 mRNA loaded LNP signal 30 seconds and 5 minutes following LNP administration in the liver and lung. Live tissues were flash frozen, and all images were acquired within 5 minutes of cryosectioning to preserve LNP localization. Phalloidin 488 was added to mounting media to label filamentous actin (F-actin) and DAPI was used to label nuclei **(i)** Confocal imaging of liver tissue 20 minutes post-LNP administration shows fluorescently labeled mRNA localized within cells, co-

stained with the capping protein eIF4E. Abundant Cy5 mRNA signal is also observed in red blood cells, suggesting LNP mRNA is associated with RBCs during circulation. Scale bars 200 uM.

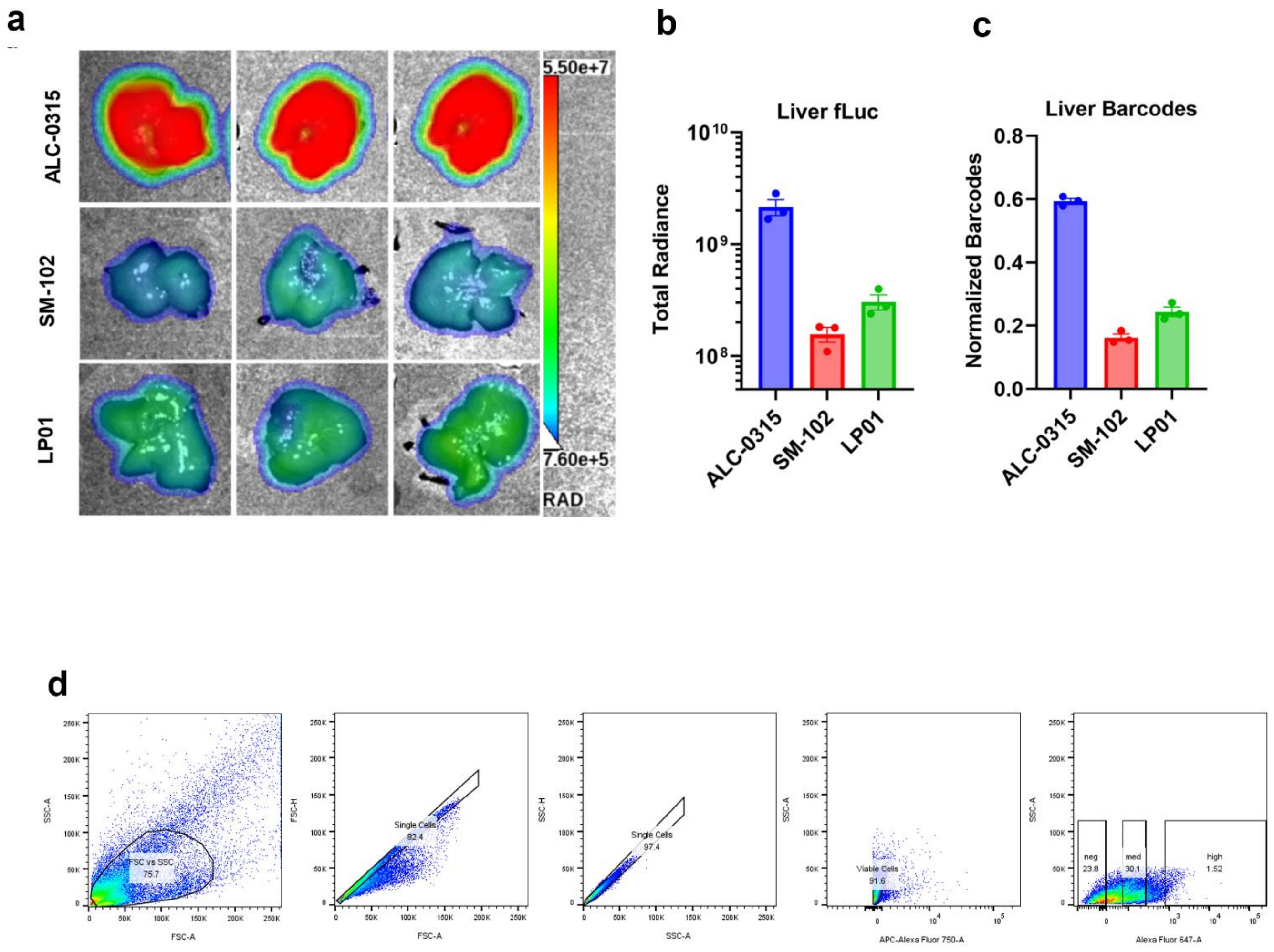

Supplementary Figure 6│ Cre mRNA barcodes recapitulate the functional activity of commercial LNPs. (a) *Ex vivo* images of mouse livers 6 hours after LNP delivery of firefly luciferase mRNA. LNPs were loaded with 0.1 mg/kg of firefly luciferase mRNA formulated using commercially available ionizable lipids (IL) and the following molar ratios of helper lipids: (IL:DSPC:Cholesterol:DMG-PEG2000); ALC-0315 (46.3:9.4:42.7:1.6), SM-102 (50:10:38.5:1.5), or LP01 (45:9:44:2). (**b**) Total radiance quantification of *ex vivo* livers showed that ALC-0315 LNPs resulted in the most luciferase protein production, followed by LP01 and SM-102 based LNPs. Mean +/- SEM (**c**) In parallel, established LNPs were loaded with Cre mRNA barcodes, pooled together, and administered to Ai14 tdTom reporter mice at 0.01 mg/kg RNA per barcode (n=3 mice) mean +/- SEM. The barcode readouts from livers collected 1-hour after multiplexed LNP administration in all three pooled mice agreed with the luminescence results obtained using individual mice. (**d**) Dot plots showing FACS gating strategy used to isolate zonated hepatocytes.

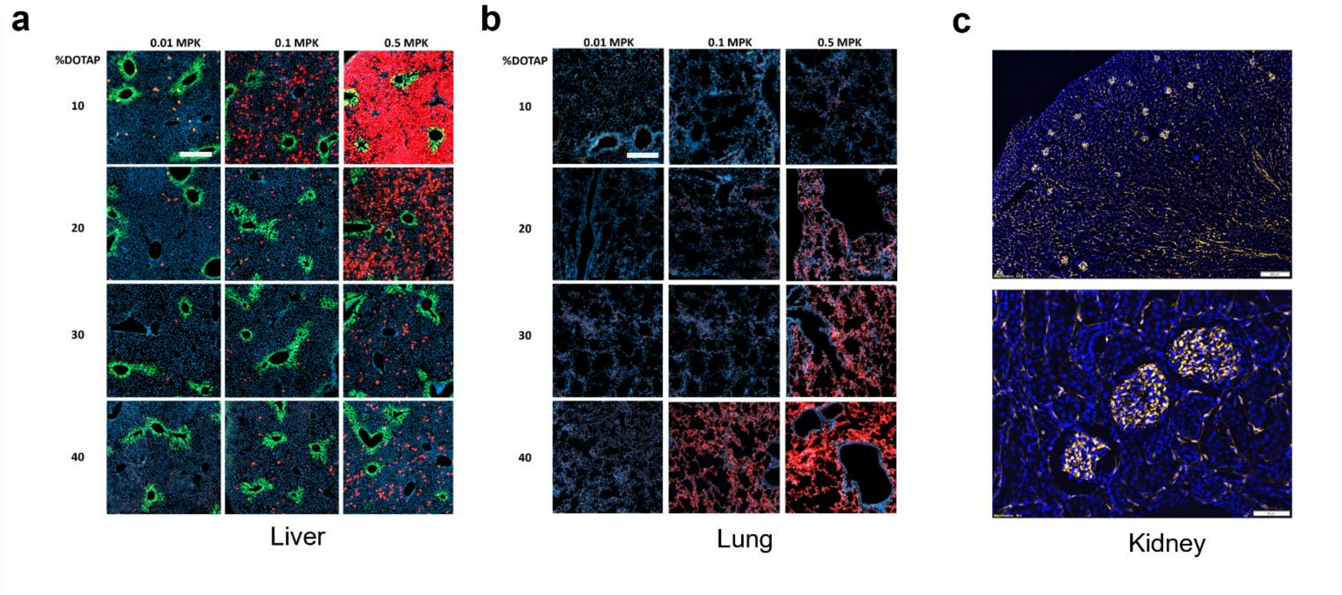

Supplementary Figure 7 │ DOTAP series dose response (a,b) Fluorescent micrographs of the livers and lungs of Ai14 mice individually administered various DOTAP LNP formulations loaded with Cre mRNA were delivered at the noted doses and collected 24h post injection. **(c)** High and low magnification images of Kidneys administered Cre mRNA encapsulated in DOTAP 40 LNPs.

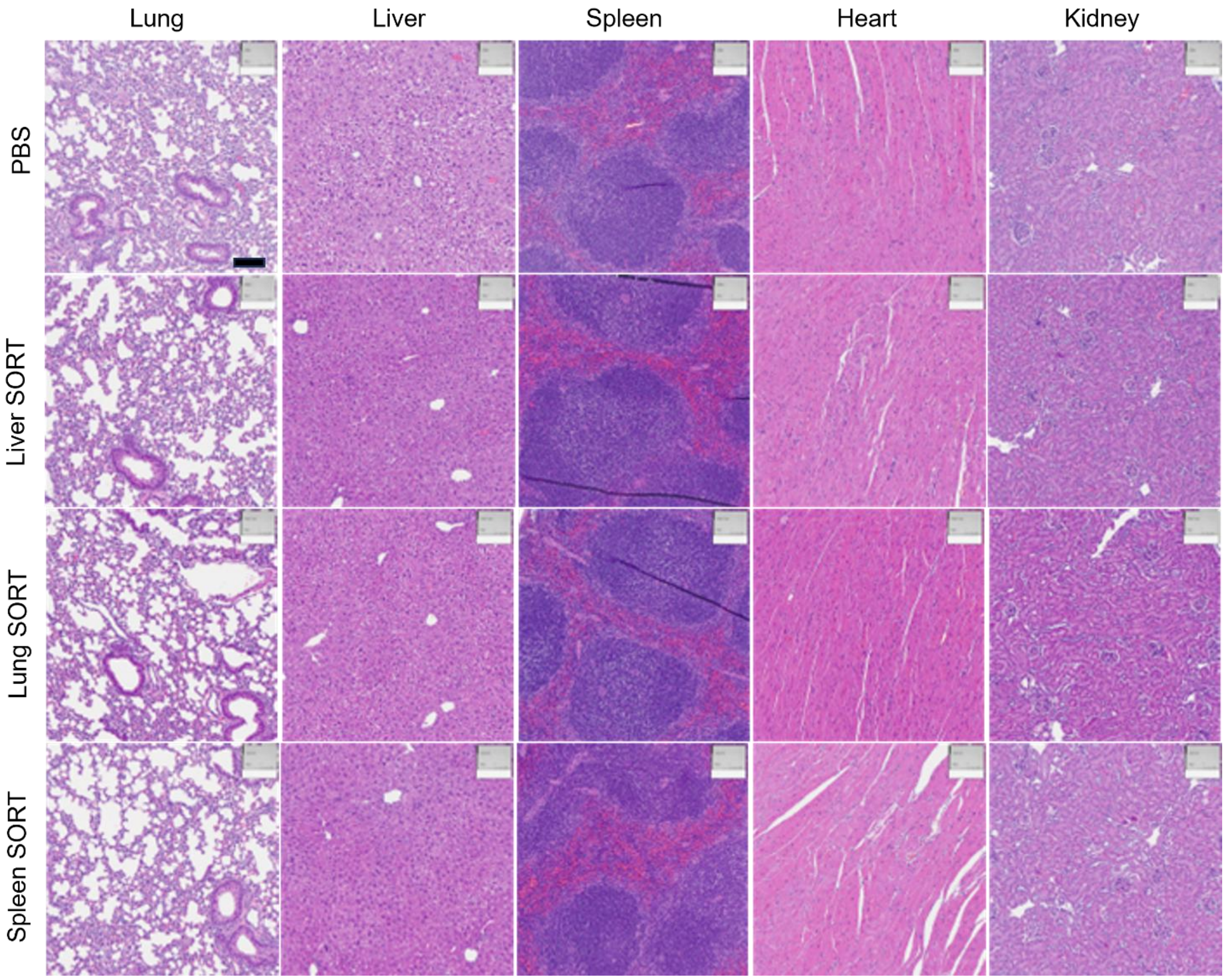

Supplementary Figure 8 │SORT LNPs are well tolerated. H&E images from major organs 24 hours after receiving 1.0 mg/kg RNA loaded in SORT LNPs. No obvious signs of injury or inflammation were apparent.

Formulation	5A2-SC8	DOPE	Chol.	PEG 2k	DOTAP	DODAP	18PA	Lipid:mRNA
Lung SORT	11.90	11.90	23.81	2.38	50	0	0	40:1
Liver SORT	19.05	19.05	38.10	3.81	0	20	0	40:1
Spleen SORT	16.67	16.67	33.33	3.33	0	0	30	40:1
DOTAP 10	21.43	21.43	42.86	4.29	10	0	0	40:1
DOTAP 20	19.05	19.05	38.10	3.81	20	0	0	40:1
DOTAP 30	16.67	16.67	33.33	3.33	30	0	0	40:1
DOTAP 40	14.29	14.29	28.57	2.86	40	0	0	40:1
	**Ion. Lipid**	**DSPC**	**Chol.**	**PEG 2k**	**DOTAP**	**DODAP**	**18PA**	**Lipid:mRNA**
ALC-0315	46.3	9.4	42.7	1.6	0	0	0	20:1
SM-102	50	10	38.5	1.5	0	0	0	20:1
LP01	45	9	44	2	0	0	0	20:1

Supplementary Table 1 │ LNP formulations.

	Liver		Lung		Spleen		Kidney	
**Tukey's multiple comparisons test**	**Summary**	**Adjusted P Value**	**Summary**	**Adjusted P Value**	**Summary**	**Adjusted P Value**	**Summary**	**Adjusted P Value**
**Liver SORT vs. DOTAP10**	**	0.0028	ns	0.9993	ns	0.9998	ns	0.8181
**Liver SORT vs. DOTAP20**	****	<0.0001	ns	0.9929	ns	0.9553	*	0.0155
**Liver SORT vs. DOTAP30**	****	<0.0001	*	0.0255	ns	0.9862	****	<0.0001
**Liver SORT vs. DOTAP40**	****	<0.0001	****	<0.0001	ns	0.9906	****	<0.0001
**Liver SORT vs. DOTAP50**	****	<0.0001	****	<0.0001	ns	0.9997	**	0.0085
**DOTAP10 vs. DOTAP20**	ns	0.5163	ns	0.8078	ns	0.9983	ns	0.2821
**DOTAP10 vs. DOTAP30**	ns	0.252	**	0.0012	ns	0.9999	***	0.0003
**DOTAP10 vs. DOTAP40**	ns	0.5764	****	<0.0001	ns	>0.9999	**	0.0013
**DOTAP10 vs. DOTAP50**	**	0.0096	****	<0.0001	ns	>0.9999	ns	0.1833
**DOTAP20 vs. DOTAP30**	ns	0.9998	ns	0.0877	ns	>0.9999	ns	0.2139
**DOTAP20 vs. DOTAP40**	ns	>0.9999	****	<0.0001	ns	>0.9999	ns	0.4785
**DOTAP20 vs. DOTAP50**	ns	0.6324	****	<0.0001	ns	0.9984	ns	>0.9999
**DOTAP30 vs. DOTAP40**	ns	0.9993	****	<0.0001	ns	>0.9999	ns	0.9997
**DOTAP30 vs. DOTAP50**	ns	0.885	****	<0.0001	ns	0.9999	ns	0.3225
**DOTAP40 vs. DOTAP50**	ns	0.5724	****	<0.0001	ns	>0.9999	ns	0.6275

Supplementary Table 2 │ Barcoding Statistics for Fig. 4b.
