# Supplementary material for: Multiplexed lipid nanoparticle barcoding reveals tissue-dynamic kinetic insights and enriched cellular tropism in hepatic zones
Source: Nat Commun. 2026 Jan 7;17:1345. doi: 10.1038/s41467-025-68103-7 (PMC12873177; doi:10.1038/s41467-025-68103-7)
Supplement: Supplementary file 2 — Reporting Summary [file 41467_2025_68103_MOESM2_ESM.pdf]

nature portfolio

Corresponding author(s): Daniel J. Siegwart

Last updated by author(s): Sep 20, 2025

Reporting Summary

Nature Portfolio wishes to improve the reproducibility of the work that we publish. This form provides structure for consistency and transparency in reporting. For further information on Nature Portfolio policies, see our Editorial Policies and the Editorial Policy Checklist.

Statistics

For all statistical analyses, confirm that the following items are present in the figure legend, table legend, main text, or Methods section.

n/a	Confirmed	
☐	☒	The exact sample size (*n*) for each experimental group/condition, given as a discrete number and unit of measurement
☐	☒	A statement on whether measurements were taken from distinct samples or whether the same sample was measured repeatedly
☐	☒	The statistical test(s) used AND whether they are one- or two-sided<br>*Only common tests should be described solely by name; describe more complex techniques in the Methods section.*
☒	☐	A description of all covariates tested
☐	☒	A description of any assumptions or corrections, such as tests of normality and adjustment for multiple comparisons
☐	☒	A full description of the statistical parameters including central tendency (e.g. means) or other basic estimates (e.g. regression coefficient) AND variation (e.g. standard deviation) or associated estimates of uncertainty (e.g. confidence intervals)
☐	☒	For null hypothesis testing, the test statistic (e.g. *F*, *t*, *r*) with confidence intervals, effect sizes, degrees of freedom and *P* value noted<br>*Give P values as exact values whenever suitable.*
☒	☐	For Bayesian analysis, information on the choice of priors and Markov chain Monte Carlo settings
☒	☐	For hierarchical and complex designs, identification of the appropriate level for tests and full reporting of outcomes
☐	☒	Estimates of effect sizes (e.g. Cohen's *d*, Pearson's *r*), indicating how they were calculated

Our web collection on statistics for biologists contains articles on many of the points above.

Software and code

Policy information about availability of computer code

Data collection	CFX Manager Version 3.1.1517.0823.<br>Zetasizer software version 7.13 (Malvern Panalytical)<br>ZEN x64 software version 1.1.0 (Carl Zeiss Microscopy GmbH)<br>LASX Version 1.4.6 28433<br>BD FACSDiva software version 8.0.1 (BD LSRFortessa)<br>Aura AMI-HTX software v.4.0.7 (Spectral Instruments Imaging)
Data analysis	ZEN blue 3.9<br>LASX Office 1.4.6 28433<br>ImageJ Version 1.54p<br>RStudio Version 2025.05.0<br>R Version 4.4.1<br>FlowJo software version 10.8.1 (FlowJo)<br>TapeStation Analysis Software 5.1<br>Graphpad Prism Version 10.2.1<br>Aura software v.4.0.7 (Spectral Instruments Imaging)

For manuscripts utilizing custom algorithms or software that are central to the research but not yet described in published literature, software must be made available to editors and reviewers. We strongly encourage code deposition in a community repository (e.g. GitHub). See the Nature Portfolio guidelines for submitting code & software for further information.

Data

Policy information about availability of data

All manuscripts must include a data availability statement. This statement should provide the following information, where applicable:

- Accession codes, unique identifiers, or web links for publicly available datasets
- A description of any restrictions on data availability
- For clinical datasets or third party data, please ensure that the statement adheres to our policy

All data needed to evaluate the conclusions in the paper are present in the Main Text and Supplementary Information. Raw data, statistics, and curve fitting parameters are included in the attached Source Data file.

Research involving human participants, their data, or biological material

Policy information about studies with human participants or human data. See also policy information about sex, gender (identity/presentation), and sexual orientation and race, ethnicity and racism.

Reporting on sex and gender	Not applicable.
Reporting on race, ethnicity, or other socially relevant groupings	Not applicable.
Population characteristics	Not applicable.
Recruitment	Not applicable.
Ethics oversight	Not applicable.

Note that full information on the approval of the study protocol must also be provided in the manuscript.

Field-specific reporting

Please select the one below that is the best fit for your research. If you are not sure, read the appropriate sections before making your selection.

☒ Life sciences ☐ Behavioural & social sciences ☐ Ecological, evolutionary & environmental sciences

For a reference copy of the document with all sections, see nature.com/documents/nr-reporting-summary-flat.pdf

Life sciences study design

All studies must disclose on these points even when the disclosure is negative.

Sample size	At least 3 replicates were used for all data unless stated otherwise. Sample size was selected based on historical experience with the models and endpoints used.
Data exclusions	No data were excluded.
Replication	The number of replicates is indicated in the figure legends; results were consistent across multiple experiments.
Randomization	This was not a randomized study.
Blinding	Researchers were not blinded

Reporting for specific materials, systems and methods

We require information from authors about some types of materials, experimental systems and methods used in many studies. Here, indicate whether each material, system or method listed is relevant to your study. If you are not sure if a list item applies to your research, read the appropriate section before selecting a response.

Materials & experimental systems

n/a	Involved in the study
☐	☒ Antibodies
☒	☐ Eukaryotic cell lines
☒	☐ Palaeontology and archaeology
☐	☒ Animals and other organisms
☒	☐ Clinical data
☒	☐ Dual use research of concern
☒	☐ Plants

Methods

n/a	Involved in the study
☒	☐ ChIP-seq
☐	☒ Flow cytometry
☒	☐ MRI-based neuroimaging

Antibodies

Antibodies used	Anti-Glutamine Synthetase ab73593<br>Anti-CD324 (E-cadherin) BioLegend Cat. No. 147307<br>Anti-eIF4E Cell signaling Cat. No. 9742
Validation	Antibodies were validated by the manufacturers. Additionally, antibody signal was compared to previous reports.

Animals and other research organisms

Policy information about studies involving animals; ARRIVE guidelines recommended for reporting animal research, and Sex and Gender in Research

Laboratory animals	Mice were housed in a barrier facility with a 12 h light/dark cycle and maintained on standard chow (2916 Teklad Global). The temperature range for the housing room is 68-79 °F (average is around 72 °F) and the humidity range is 30-50% (average is around 50%). For tdTomato reporter mice transfection analysis, male and female B6.Cg-Gt(ROSA)26Sortm14(CAG-tdTomato)Hze/J mice (also known as Ai14 mice, 6-8 weeks) housed in UTSW animal facility were used.
Wild animals	The study did not involve the use of wild animals.
Reporting on sex	Both male and female mice were used. Experimental group contained co-equal proportions of male and female mice.
Field-collected samples	The study did not involve samples collected from the field.
Ethics oversight	Animal experiments were approved by the Institution Animal Care and Use Committee of The University of Texas Southwestern Medical Center under APN 2015-101118-G, APN 2015-100933, APN 2016-101430, and APN 2017-102283 and were consistent with local, state, and federal regulations as applicable.

Note that full information on the approval of the study protocol must also be provided in the manuscript.

Plants

Seed stocks	Not applicable.
Novel plant genotypes	Not applicable.
Authentication	Not applicable.

Flow Cytometry

Plots

Confirm that:

☒ The axis labels state the marker and fluorochrome used (e.g. CD4-FITC).

☒ The axis scales are clearly visible. Include numbers along axes only for bottom left plot of group (a 'group' is an analysis of identical markers).

☒ All plots are contour plots with outliers or pseudocolor plots.

☒ A numerical value for number of cells or percentage (with statistics) is provided.

Methodology

Sample preparation	Mouse tissues were collected and immediately placed in ice-cold PBS. For the liver and lungs, a small part of the tissues was cut into small pieces and further digested in 1× tissue digestion media (RPMI supplemented with 2% wt:vol BSA, 300 U/mL collagenase, and 100 U/mL hyaluronidase) at 37 °C for 45 min to 1 h. After incubation, the undigested fragments were further ground and filtered through a 70-µm cell strainer together with the digested cell suspension. For the spleen and lymph nodes, tissues were directly minced and filtered through 70-µm cell strainers. After centrifuged and removed of the supernatant, the cell pellets were resuspended in red blood cell lysis buffer (BioLegend, 420301) for 5-10 min at room temperature (cell suspensions from the lymph nodes were not removed of red blood cells). Then the cell staining buffer (BioLegend) was added and the cell suspensions were filtered and centrifuged again. The resuspended cells were pre-incubated with mouse Fc-receptor blocker (BioLegend) and then stained with fluorophore-conjugated antibodies at 4 °C in dark for 30 min. Flow data were collected using BD LSR Fortessa flow cytometer and analyzed using FlowJo software version 10.8.1.
Instrument	BD FACSDiva software version 8.0.1 (BD LSRFortessa)
Software	FlowJo software version 10.8.1 (FlowJo)
Cell population abundance	We used toTomato Ai14 mice to evaluate tissue specific Cre recombinase-mediated recombination
Gating strategy	Gates for TdTom+ in cells were drawn based on control mice. Gates for CD324 were drawn based on previous reports. Gating strategy were provided in figures of Supplementary Information.

☒ Tick this box to confirm that a figure exemplifying the gating strategy is provided in the Supplementary Information.
